# Association of 25-Hydroxyvitamin D with Preterm Birth and Premature Rupture of Membranes: A Mendelian Randomization Study

**DOI:** 10.3390/nu15163593

**Published:** 2023-08-16

**Authors:** Haoyue Cheng, Peihan Chi, Yan Zhuang, Xialidan Alifu, Haibo Zhou, Yiwen Qiu, Ye Huang, Libi Zhang, Diliyaer Ainiwan, Zhicheng Peng, Shuting Si, Hui Liu, Yunxian Yu

**Affiliations:** 1 Department of Public Health and Department of Anesthesiology, the Second Affiliated Hospital of Zhejiang University School of Medicine, Hangzhou 310009, China; 3150101365@zju.edu.cn (H.C.);; 2 Department of Epidemiology & Health Statistics, School of Public Health, School of Medicine, Zhejiang University, Hangzhou 310058, China; 3 Yiwu Maternity and Children Hospital, Yiwu 322000, China; 4 Sir Run Run Shaw Hospital, School of Medicine, Zhejiang University, Hangzhou 310000, China

**Keywords:** 25-hydroxyvitamin D, Mendelian randomization, preterm birth, premature rupture of membranes

## Abstract

Low vitamin D (VitD) level is a risk factor for preterm birth (PTB), but the results of previous studies remained inconsistent, which may be influenced by the confounding factors and different types of PTB. We performed Mendelian randomization (MR) to uncover the association of 25-hydroxyvitamin D (25(OH)D) with PTB, premature rupture of membranes (PROM), and preterm premature rupture of membranes (PPROM). This study was conducted in Zhoushan Maternal and Child Health Hospital, Zhejiang, from August 2011 to March 2022. Plasma 25(OH)D levels in three trimesters of pregnancy were measured. We conducted an MR analysis utilizing a genetic risk score (GRS) approach, which was based on VitD-associated single-nucleotide polymorphisms. The prospective cohort study included 3923 pregnant women. The prevalence of PTB, PROM, and PPROM were 6.09%, 13.18%, and 1.33%, respectively. Compared to those without vitamin D deficiency (VDD), only vaginally delivering pregnant women with VDD had a 2.69 (1.08–6.68) times risk of PTB. However, MR analysis did not support the association. One-unit higher GRS was not associated with an increased risk of PTB, regardless of the trimesters (OR [95% CI]: 1.01 [0.93–1.10], 1.06 [0.96–1.18], and 0.95 [0.82–1.10], respectively). When further taking PROM and PPROM as the outcomes, the MR analysis also showed no consistent evidence of a causal effect of VitD levels on the risk of them. Our MR analyses did not support a causal effect of 25(OH)D concentrations in the three trimesters on PTB, PROM, and PPROM.

## 1. Introduction

Preterm birth (PTB) is defined as birth before 37 completed weeks of gestation or fewer than 259 days from the first date of a woman’s last menstrual period [1]. The preceding obstetric factors that contribute to PTB include: (1) medically indicated delivery; (2) spontaneous onset of preterm labor with intact membranes; and (3) preterm premature rupture of membranes (PPROM) [2]. For neonates, PTB is a risk factor that exerts long-term implications on health, well-being, and overall development in later stages of life. Based on 2010 data from 184 countries, roughly 15 million infants are born prematurely globally, accounting for an approximate global PTB rate of 11% [3]. In China, the PTB rate increased from 5.9% in 2012 to 6.4% in 2018 (annual rate of increase 1.3, 95% CI: 0.6–2.1) [4]. And, this situation has also occurred in many other countries [5].

The related factors of PTB include maternal age [6], PTB history [7], infection [8], maternal nutritional status [9], and so on. In recent years, there have been widespread concerns regarding the correlation between vitamin D deficiency (VDD) during pregnancy and an increased risk of PTB [10]. VDD is common in different populations and is particularly notable in pregnant women [11]. Serum 25-hydroxyvitamin D (25(OH)D) is widely recognized as a biomarker for assessing vitamin D (VitD) levels in circulation among humans. However, the protective effect of maternal 25(OH)D during pregnancy on the occurrence of PTB remains controversial [12,13,14]. A meta-analysis conducted in 2017 revealed variations in the association between vitamin D and different types of preterm birth [12]. Nevertheless, there is limited research exploring the association between VitD levels in pregnant women and the occurrence of premature rupture of membranes (PROM), which is one of the main causes of PTB.

Studies on genetic variants that specifically affect the 25(OH)D concentration may provide valuable insights into clarifying the causal association of VitD with PTB and PROM. Therefore, we intend to utilize the Mendelian analysis (MR) to establish the association between VitD and the above outcomes. MR analysis employs genetic variants as instrumental variables (IVs) to investigate the causality of an association, as the assignment of genes during conception is random at the population level and thus unaffected by confounding factors [15]. Advances in the methodology of large-scale genetic association studies have identified some single-nucleotide polymorphisms (SNPs) from different genes that influence the 25(OH)D concentration [16,17]. The biotransformation gene *CYP3A4* affects the synthesis of 25(OH)D; the transport gene *GC* encodes the vitamin D-binding protein; and the catabolism gene *CYP24A1* is associated with the clearance of 25(OH)D [18].

In this study, a prospective cohort study design was used to investigate the causal effect of 25(OH)D concentrations in the three trimesters on PTB, PROM, and PPROM using genetic variants that are associated with the 25(OH)D concentrations as IVs in a Mendelian randomization analysis.

## 2. Materials and Methods

### 2.1. Study Population

The participants were from a prospective cohort study in Zhoushan, China, from August 2011 to March 2022. The Zhoushan Pregnant Women Cohort (ZPWC) study was described in the previous study [18]. Based on the inclusion and exclusion criteria established in the ZPWC study, we further included women who had plasma 25(OH)D concentrations measured in the first, second, or third trimester while excluding those who lacked delivery information, experienced post-term births, stillbirths, or multiple pregnancies. A subset of 3923 pregnant women was selected for this study.

Information on sociodemographic characteristics, lifestyle, and personal health status was collected by trained interviewers during a face-to-face interview. Aside from that, blood samples for the full cohort were collected and stored for biochemical tests and genotyping. The study protocol was approved by the Research Ethics Committee of Zhoushan Maternal and Child Health Hospital and Zhejiang University School of Medicine.

### 2.2. Plasma 25(OH)D Measurement

According to the VitD testing gold standard established by the National Institute of Standards and Technology (NIST) in the United States [19], the plasma concentrations of 25-hydroxyvitamin D2 (25(OH)D_2_) and 25-hydroxyvitamin D3 (25(OH)D_3_) in the first, second, or third trimester of pregnancy were measured using liquid chromatography-tandem mass spectrometry (LC-MS ACQUITYUPLC-TQD; Waters Corporation, Milford, MA, USA), and their sum represented the total 25(OH)D concentrations.

Based on the Endocrine Society’s clinical practice guideline [20], the 25(OH)D concentrations were classified into three categories: VitD deficiency (<20 ng/mL), VitD insufficiency (20–30 ng/mL), and VitD sufficiency (≥30 ng/mL). Previous research has demonstrated a significant biological effect associated with 25(OH)D levels below 20 ng/mL [10]. Hence, we classified VitD into two groups in the follow-up analysis: VitD deficiency (<20 ng/mL), and VitD non-deficiency (≥20 ng/mL).

### 2.3. SNP Selection and Genotyping

The previous study reported the criteria for candidate genes and SNPs related to the synthesis, transport, and catabolism of vitamin D [18]. We first selected *NADSYN1/DHCR7* (rs1790349 and rs12785878), *GC* (rs222040, rs1155563, rs16846876, rs2298849, rs7041, and rs4588), *CYP24A1* (rs2209314), *CYP2R1* (rs10741657), and *VDR* (rs2228570, rs7975232, and rs757343) as candidate SNPs. Based on the Hardy–Weinberg equilibrium (*r*^2^ > 0.8) and the genotype success rate (>95%), five transport SNPs (*GC*-rs1155563, *GC*-rs16846876, *GC*-rs2298849, *GC*-rs7041, and *GC*-rs4588), and one catabolism SNP (*CYP24A1*-rs2209314) were selected. SNP genotyping was performed using the Sequenom MassARRAY iPLEX Gold platform (Sequenom, San Diego, CA, USA).

### 2.4. GRSs

We conducted a one-sample MR analysis utilizing a genetic risk score (GRS) approach. Based on the correlation between the above SNPs and the 25(OH)D concentrations, we adopted an additive genetic model. Genotypes containing 0, 1, or 2 alleles were assigned scores of 0, 1, or 2, respectively. GRSs were the sum of scores for each SNP multiplied by the unweighted value.

### 2.5. Definition of the Outcomes

All outcomes were collected from the Maternal and Child Health Information Management System of Zhoushan Maternal and Child Health Hospital. PTB is defined as births prior to 37 completed weeks of gestation [1]. PROM refers to the rupture or breaking of the amniotic sac and leakage of amniotic fluid before the onset of labor. When PROM occurs before 37 completed weeks of gestation, it was defined as PPROM. Finally, spontaneous PTB refers to PTB without any medical or surgical intervention to initiate or facilitate the birth.

### 2.6. Statistical Analysis

The descriptive statistics for continuous variables were reported as mean ± standard deviation (SD), while categorical variables were presented as frequency and percentage. ANOVA and the χ^2^ test were used for continuous variables and categorical variables, respectively, to compare the characteristics between groups (Healthy, only PTB, only PROM, PPROM). Aside from that, the 25(OH)D concentrations, gestational week at blood sampling and sampling season measured in the first, second, or third trimester of pregnancy were also described.

Initially, linear regression was used to examine the association between each SNP and the 25(OH)D concentrations in different trimesters, assuming a linear effect of each VitD-related SNP for each additional allele on 25(OH)D. The Cragg–Donald F-statistic was used to estimate the strength of the association, with F values greater than 10 considered adequate for MR analysis [21]. Additionally, the potential effects of pleiotropy for the above six SNPs were examined using MR–Egger regression, where the *p*-value of the intercept provides a valid test of directional pleiotropy [22].

There were three outcomes in this study: PTB, PROM, and PPROM. The direct effect of VitD in three trimesters on the risk for PTB, PROM, and PPROM was assessed using multivariable logistic regression models. The effect of SNPs on the risk for PTB, PROM, and PPROM was respectively assessed using multivariable logistic regression models. We performed a one-sample MR analysis using two-stage least-squares (2SLS) regression using the ivreg command from the ivreg package in R. GRS that was used as IV to estimate the causal effect of 25(OH)D on PPROM, PROM, and PTB, and the effect estimates were presented per 5 ng/mL increase in the 25(OH)D concentrations or VitD deficiency. Models were adjusted for the following potential confounders: maternal age, pre-pregnancy BMI, educational level, parity, PTB history/gestational age, gestational week at blood sampling, and sampling season. 

To exclude medically indicated preterm birth caused by complications such as gestational hypertension, further analysis was restricted to pregnant women who delivered vaginally. This was conducted to determine a clear association between VitD and spontaneous PTB. All statistical analyses were performed using R 4.2.2 with statistical significance at *p* < 0.05.

## 3. Results

### 3.1. Participant Characteristics

The demographic characteristics and maternal health status during pregnancy were compared among the Healthy, only PTB, only PROM, and PPROM groups (Table 1). The prevalence of PTB, PROM, and PPROM in the 3923 participants was 6.09%, 13.18%, and 1.33%, respectively. PTB women without PROM had the highest pre-pregnancy BMI and prevalence of cesarean section and PTB history. No significant difference in maternal age, educational level, gravidity, and parity was observed among the four groups.

The mean 25(OH)D concentrations were 18.01 ± 8.17, 27.44 ± 10.57, and 28.57 ± 11.98 ng/mL in three trimesters, respectively. Despite the fact that the 25(OH)D concentrations increased as gestational weeks progressed, VDD remained prevalent among pregnant women. The prevalence of VDD was 64.56%, 26.30%, and 27.05% in three trimesters, respectively. 25(OH)D levels in the first and second trimesters were not significantly different among Healthy, only PTB, only PROM, and PPROM groups (Table 2). In the third trimester, PROM women without PTB had the highest 25(OH)D concentration and the lowest prevalence of VDD.

### 3.2. Association of 25(OH)D Level with PTB

Table S1 presented the association of 25(OH)D concentrations in the three trimesters with PTB in pregnant women. There was no association between VitD levels and the risk of PTB. However, when restricted to pregnant women who delivered vaginally, there was a higher risk of PTB among those with VDD in the third trimester of pregnancy (Table S2). Compared to those without VDD, vaginally delivering pregnant women with VDD had a 2.69 (1.08–6.68) times risk of PTB.

Table 3 showed the causal coefficients from the MR analysis for the association of PTB with VitD-determined GRS. However, one-unit higher GRS was not associated with an increased risk of PTB, regardless of the trimesters (OR [95% CI]: 1.01 [0.93–1.10] in the first trimester, 1.06 [0.96–1.18] in the second trimester, and 0.95 [0.82–1.10] in the third trimester, respectively). The above results remained consistent among pregnant women who delivered vaginally (Table S3).

### 3.3. Association of 25(OH)D Levels with PROM

When PROM was considered as the outcome, in the crude model, only the 25(OH)D concentration in the third trimester was found to be associated with PROM (Table S4). However, after adjusting for covariates, there was no significant association between them. The MR analysis also showed no consistent evidence of a causal effect of VitD levels on the risk of PROM (Table 4). The odds ratio for PROM was 1.05 (95% CI: 0.99–1.12), 1.01 (95% CI: 0.95–1.08), and 1.06 (95% CI: 1.00–1.12) per 5 ng/mL increase in the 25(OH)D concentrations in three trimesters, respectively.

### 3.4. Association of 25(OH)D Levels with PPROM

Taking into account the different classifications of PTB, we also conducted an analysis specifically focusing on PPROM. We did not find evidence of any association between 25(OH)D levels and PPROM in the multivariable analysis (Table S5). The MR analysis also showed no consistent evidence of a causal effect of VitD levels on the risk of PPROM (Table 5). The odds ratio for PPROM was 1.10 (95% CI: 0.94–1.30), 1.05 (95% CI: 0.86–1.29), and 0.99 (95% CI: 0.75–1.30) per 5 ng/mL increase in the 25(OH)D concentrations in three trimesters, respectively.

## 4. Discussion

Previous studies found that the VitD levels might influence birth outcomes as well as maternal and child health, such as PTB and PROM, but the results of the studies remained inconsistent, and the causality of the association has been uncertain. In our study, no evidence was found of the association of 25(OH)D concentrations in the three trimesters with PTB, PROM, or PPROM in the one-sample MR analysis, nor did we find any appreciable evidence of a causal effect of 25(OH)D concentrations that are less than 20 ng/mL on the risk of the outcomes.

Currently, there is a lack of consensus in the study results regarding the association between VitD levels in three trimesters and the risk of PTB. In a prospective cohort study involving 2327 pregnant women, it was observed that a low maternal serum concentration of 25(OH)D (<20 ng/mL) before 20 weeks of gestation significantly elevated the incidence of PTB (OR = 1.8, 95% CI: 1.3–2.6). This association remained consistent when considering cases that were medically indicated or occurred spontaneously [23]. However, the results from another prospective cohort study in Europe did not provide support for an association between maternal first-trimester VDD (<30 ng/mL) and the risk of PTB [24]. And our previous studies also did not find a significant association between them [10]. A study in China even found a reverse association between VDD in the second trimester and the risk of PTB (OR = 1.04, 95% CI: 1.02–1.06) [25].

Our previous study has indicated that the 25(OH)D concentrations increased notably with gestational week [26]. Therefore, it is critical to differentiate the association between VitD levels and PTB across the different trimesters of pregnancy. Our study found that only VD status in the third trimester was associated with PTB in pregnant women undergoing vaginal delivery. Wagner et al. [27] also found that women with 25(OH)D levels greater than 40 ng/mL had a 47% lower risk of PTB compared to those with 25(OH)D levels below 40 ng/mL. In the aforementioned analysis on the association of VitD with PTB, there were variations in the gestational weeks at which VitD was measured [23,24,25]. Therefore, the association between them may differ across the different trimesters of pregnancy. Further studies are needed to validate this hypothesis.

Considering possible risk factors for PTB, including adverse lifestyle, psychological stress, younger or older age during pregnancy, and so on, the inconsistency among different studies may be influenced by various confounding factors [28]. Therefore, using genetic variants as instrumental variables to reduce the possibility of confounding is critical. To our knowledge, this is the first study to examine the association between VitD and PTB using MR analysis. We found that there was a null association between genetically determined 25(OH)D concentrations in the three trimesters and PTB, which is consistent with our previous observational study [10]. Aside from that, our study found that VDD in the third trimester increased the risk of spontaneous PTB (OR = 2.69, 95% CI: 1.08–6.68). However, when using MR analysis, the association of VitD with spontaneous PTB was not significant. The difference between these two results further confirms the necessity of utilizing MR analysis.

In addition to the influence of confounding factors, the diverse pathogenesis of PTB may also contribute to potential variations in the association between VitD and different types of PTB [12]. PROM accounts for 30% of PTB [29]. Therefore, we also investigated the causal effect of 25(OH)D concentrations in the three trimesters on PROM and PPROM. The main causes of PPROM are infection and inflammation. The previous study has found that when pregnant women experience acute chorioamnionitis, there is an increase in inflammation-related proteases, the activation of cytokines, and a significant reduction in the tensile strength and elasticity of the fetal membranes, leading to membrane rupture and subsequent preterm birth [30,31]. It can not be ignored that VitD plays an important role in regulating immune responses [32]. Therefore, we speculated that vitamin D levels might be associated with PPROM. However, there were only a few studies that analyzed the association between VitD levels and PROM. Ni et al. [33] found that 25(OH)D status in the first trimester did not influence the incidence rate of PROM in Chinese pregnant women. Results from a prospective observational study also showed that the incidence rate of PROM was not significantly different between the three groups (≤20 ng/mL, 21–29 ng/mL, and ≥30 ng/mL) [25]. Our study confirmed that there was no association between the 25(OH)D concentrations and PROM whether using regression models or MR analysis. When we further combined the outcomes of PROM and PTB, we also did not find any significant association between genetically determined VitD and PPROM. Additionally, even when considering only pregnant women who delivered vaginally, the association between VitD and PTB remained non-significant. These results suggested that the negative association between 25(OH)D concentrations in the three trimesters and PTB was not influenced by the type of PTB. However, considering that this is the first study analyzing the association of VitD with PTB, PROM, and PPROM using a one-sample MR analysis, further similar studies are needed to provide additional support.

Our study is characterized by several notable strengths, with one being the utilization of genetic variants as IVs to mitigate the potential impact of confounding factors. In addition, as VitD levels fluctuate throughout pregnancy, we assessed the 25(OH)D concentrations in three trimesters, which was less commonly observed in previous studies. Some limitations of our study should also be noted. First, the cases of the PPROM were small, which limited the credibility of the results. More larger sample size studies are needed to analyze the association between VitD and the different types of PTB. Second, the composition of women with plasma 25(OH)D concentrations in the first, second, and third trimesters was not identical, which could potentially introduce selection bias. Third, the absence of 25(OH)D GWAS data in Asian populations, which would facilitate the construction of an ethnicity-specific GRS, restricts the application of MR. Additionally, the six identified SNPs could only account for approximately 2–3% of the variation in the 25(OH)D phenotype.

## 5. Conclusions

In conclusion, our MR analyses did not support a causal effect of 25(OH)D concentrations in the three trimesters on PTB. When further considering the association between the different types of PTB and VitD, there was still no evidence to support the association of VitD with PROM and PPROM.

## Figures and Tables

**Table 1:** Characteristics of study participants (*n* = 3923).

Variables	Healthy Group (*n* = 3219)	Only PTB Group (*n* = 187)	Only PROM Group (*n* = 465)	PPROM Group (*n* = 52)	*p*
Maternal age, years	29.31 ± 3.88	29.74 ± 3.95	29.40 ± 3.94	29.67 ± 4.64	0.460
Pre-pregnancy BMI, kg/m^2^	21.10 ± 2.72	21.85 ± 2.96	21.32 ± 2.73	21.81 ± 2.85	<0.001
Educational level					0.198
Junior high school or below	244 (7.58)	20 (10.70)	35 (7.53)	6 (11.54)	
High school	574 (17.83)	33 (17.65)	65 (13.98)	11 (21.15)	
College or above	2401 (74.59)	134 (71.66)	365 (78.49)	35 (67.31)	
Gravidity					0.168
1	1502 (46.66)	86 (45.99)	232 (49.89)	29 (55.77)	
2	904 (28.08)	49 (26.20)	134 (28.82)	6 (11.54)	
3	499 (15.50)	31 (16.58)	55 (11.83)	11 (21.15)	
≥4	314 (9.75)	21 (11.23)	44 (9.46)	6 (11.54)	
Parity					0.278
Primipara	2383 (74.03)	145 (77.54)	360 (77.42)	41 (78.85)	
Multipara	836 (25.97)	42 (22.46)	105 (22.58)	11 (21.15)	
Delivery mode					<0.001
Vaginal delivery	1799 (55.89)	72 (38.50)	346 (74.41)	32 (61.54)	
Cesarean	1420 (44.11)	115 (61.50)	119 (25.59)	20 (38.46)	
PTB history					0.003
No	3173 (98.57)	178 (95.19)	456 (98.06)	50 (96.15)	
Yes	46 (1.43)	9 (4.81)	9 (1.94)	2 (3.85)	

Abbreviations: PTB, preterm birth; PROM, premature rupture of membranes; PPROM, preterm premature rupture of membranes; BMI, body mass index.

**Table 2:** Comparison of 25(OH)D concentrations and related information in three trimesters among different groups.

Variables	Healthy Group	Only PTB Group	Only PROM Group	PPROM Group	*p*
**First trimester (*n* = 3626)**					
25(OH)D, ng/mL	17.92 ± 8.13	17.87 ± 7.81	18.51 ± 8.39	19.58 ± 10.06	0.284
Vitamin D deficiency					0.294
No	1040 (34.89)	61 (34.27)	164 (39.33)	20 (40.00)	
Yes	1941 (65.11)	117 (65.73)	253 (60.67)	30 (60.00)	
Gestational week at blood sampling	11.91 ± 0.90	11.78 ± 0.96	11.98 ± 0.75	12.08 ± 0.79	0.052
Sampling season					0.565
Summer/Autumn	1513 (50.75)	95 (53.37)	221 (53.00)	29 (58.00)	
Spring/Winter	1468 (49.25)	83 (46.63)	196 (47.00)	21 (42.00)	
**Second trimester (*n* = 1840)**					
25(OH)D, ng/mL	27.39 ± 10.55	28.13 ± 10.30	27.49 ± 10.55	28.01 ± 13.00	0.943
Vitamin D deficiency					0.883
No	1097 (73.87)	50 (74.63)	191 (72.08)	18 (78.26)	
Yes	388 (26.13)	17 (25.37)	74 (27.92)	5 (21.74)	
Gestational week at blood sampling	24.10 ± 3.51	23.89 ± 3.41	23.92 ± 3.41	24.34 ± 2.33	0.830
Sampling season					0.306
Summer/Autumn	766 (51.58)	31 (46.27)	141 (53.21)	8 (34.78)	
Spring/Winter	719 (48.42)	36 (53.73)	124 (46.79)	15 (65.22)	
**Third trimester (*n* = 2044)**					
25(OH)D, ng/mL	28.33 ± 12.10	25.07 ± 12.82	30.81 ± 10.75	27.65 ± 11.23	0.007
Vitamin D deficiency					0.012
No	1270 (72.41)	18 (54.55)	195 (79.27)	8 (72.73)	
Yes	484 (27.59)	15 (45.45)	51 (20.73)	3 (27.27)	
Gestational week at blood sampling	33.42 ± 3.73	31.13 ± 3.59	34.39 ± 3.40	32.51 ± 3.87	<0.001
Sampling season					0.303
Summer/Autumn	802 (45.72)	17 (51.52)	127 (51.63)	6 (54.55)	
Spring/Winter	952 (54.28)	16 (48.48)	119 (48.37)	5 (45.45)	

**Table 3:** Association of 25(OH)D concentrations in three trimesters with PTB from MR analysis.

Variables	*n* (%)	Model 1 *		Model 2 †	
		OR (95% CI)	*p*	OR (95% CI)	*p*
**First trimester**					
25(OH)D, ng/mL	228 (6.29)	1.00 (0.95, 1.05)	0.979	1.00 (0.96, 1.05)	0.945
Vitamin D deficiency					
No	81 (6.30)	ref.	-	ref.	-
Yes	147 (6.28)	1.00 (0.79, 1.27)	0.979	0.99 (0.80, 1.23)	0.945
**Second trimester**					
25(OH)D, ng/mL	90 (4.89)	1.01 (0.97, 1.05)	0.700	1.01 (0.97, 1.06)	0.636
Vitamin D deficiency					
No	68 (5.01)	ref.	-	ref.	-
Yes	22 (4.55)	0.95 (0.75, 1.21)	0.701	0.94 (0.72, 1.22)	0.637
**Third trimester**					
25(OH)D, ng/mL	44 (2.15)	1.01 (0.99, 1.04)	0.261	1.01 (0.99, 1.03)	0.326
Vitamin D deficiency					
No	26 (1.74)	ref.	-	ref.	-
Yes	18 (3.25)	0.91 (0.76, 1.08)	0.270	0.93 (0.80, 1.08)	0.332

* Model 1: Crude model. † Model 2: Adjusted for maternal age, pre-pregnancy BMI, educational level, parity, PTB history, gestational week at blood sampling, and sampling season.

**Table 4:** Association of 25(OH)D concentrations in three trimesters with PROM from MR analysis.

Variables	*n* (%)	Model 1 *		Model 2 †	
		OR (95% CI)	*p*	OR (95% CI)	*p*
**First trimester**					
25(OH)D, ng/mL	467 (12.88)	1.01 (0.94, 1.08)	0.784	1.01 (0.95, 1.08)	0.690
Vitamin D deficiency					
No	184 (14.32)	ref.	-	ref.	-
Yes	283 (12.09)	0.96 (0.69, 1.32)	0.784	0.94 (0.70, 1.26)	0.691
**Second trimester**					
25(OH)D, ng/mL	288 (15.65)	1.01 (0.95, 1.09)	0.687	1.02 (0.95, 1.10)	0.641
Vitamin D deficiency					
No	209 (15.41)	ref.	-	ref.	-
Yes	79 (16.32)	0.92 (0.62, 1.37)	0.688	0.90 (0.58, 1.40)	0.642
**Third trimester**					
25(OH)D, ng/mL	257 (12.57)	1.00 (0.95, 1.06)	0.939	1.01 (0.96, 1.06)	0.827
Vitamin D deficiency					
No	203 (13.62)	ref.	-	ref.	-
Yes	54 (9.76)	0.99 (0.68, 1.43)	0.939	0.96 (0.69, 1.35)	0.827

* Model 1: Crude model. † Model 2: Adjusted for maternal age, pre-pregnancy BMI, educational level, parity, gestational age, gestational week at blood sampling, and sampling season.

**Table 5:** Association of 25(OH)D concentrations in three trimesters with PPROM from MR analysis.

Variables	*n* (%)	Model 1 *		Model 2 †	
		OR (95% CI)	*p*	OR (95% CI)	*p*
**First trimester**					
25(OH)D, ng/mL	50 (1.38)	1.00 (0.97, 1.02)	0.795	1.00 (0.98, 1.02)	0.863
Vitamin D deficiency					
No	20 (1.56)	ref.	-	ref.	-
Yes	30 (1.28)	1.01 (0.91, 1.14)	0.796	1.01 (0.91, 1.12)	0.863
**Second trimester**					
25(OH)D, ng/mL	23 (1.25)	1.00 (0.98, 1.02)	0.937	1.00 (0.98, 1.02)	0.989
Vitamin D deficiency					
No	18 (1.33)	ref.	-	ref.	-
Yes	5 (1.03)	1.00 (0.89, 1.13)	0.937	1.00 (0.88, 1.14)	0.989
**Third trimester**					
25(OH)D, ng/mL	11 (0.54)	1.00 (0.99, 1.02)	0.634	1.00 (0.99, 1.01)	0.697
Vitamin D deficiency					
No	8 (0.54)	ref.	-	ref.	-
Yes	3 (0.54)	0.98 (0.90, 1.06)	0.635	0.99 (0.91, 1.06)	0.698

* Model 1: Crude model. † Model 2: Adjusted for maternal age, pre-pregnancy BMI, educational level, parity, PTB history, gestational week at blood sampling, and sampling season.

## Data Availability

The data presented in this study are available on request from the corresponding author. The data are not publicly available because they contain information that could compromise the privacy of the research participants.

## Supplementary Materials

The following supporting information can be downloaded at: https://www.mdpi.com/article/10.3390/nu15163593/s1, Table S1: Association of 25(OH)D concentrations in three trimesters with PTB; Table S2: Association of 25(OH)D concentrations in three trimesters with spontaneous PTB; Table S3: Association of 25(OH)D concentrations in three trimesters with spontaneous PTB from MR analysis; Table S4: Association of 25(OH)D concentrations in three trimesters with PROM; Table S5: Association of 25(OH)D concentrations in three trimesters with PPROM; Table S6: Association of single SNP with PTB; Table S7: Association of single SNP with spontaneous PTB; Table S8: Association of single SNP with PROM; and Table S9: Association of single SNP with PPROM.
