# Supplementary material for: Association of 25-Hydroxyvitamin D with Preterm Birth and Premature Rupture of Membranes: A Mendelian Randomization Study
Source: Nutrients. 2023 Aug 16;15(16):3593. doi: 10.3390/nu15163593 (PMC10459690; doi:10.3390/nu15163593)
Supplement: Supplementary file 1 [file nutrients-15-03593-s001.zip › nutrients-2550507-supplementary.pdf]

Association of 25-hydroxyvitamin D with preterm birth and premature rupture of membranes: a Mendelian randomization study

Haoyue Cheng [1,2], Peihan Chi [1,2], Yan Zhuang [1,2], Xialidan Alifu [1,2], Haibo Zhou [1,2], Yiwen Qiu [1,2], Ye Huang [1,2], Libi Zhang [1,2], Diliyaer Ainiwan [1,2], Zhicheng Peng [1,2], Shuting Si [3], Hui Liu [4] and Yunxian Yu [1,2,*]

[1] Department of Public Health and Department of Anesthesiology, the Second Affiliated Hospital of Zhejiang University School of Medicine, Hangzhou 310009, China.
[2] Department of Epidemiology & Health Statistics, School of Public Health, School of Medicine, Zhejiang University, Hangzhou 310058, China.
[3] Yiwu Maternity and Children Hospital, Yiwu 322000, China.
[4] Sir Run Run Shaw Hospital, School of Medicine, Zhejiang University, Hangzhou 310000, China.
* Correspondence: yunxianyu@zju.edu.cn

Table S1. Association of 25(OH)D concentrations in three trimesters with PTB

Variables	Model 1*		Model 2†	
	OR (95% CI)	*p*	OR (95% CI)	*p*
**First trimester**				
25(OH)D, ng/mL	1.02 (0.94, 1.10)	0.662	1.01 (0.93, 1.10)	0.792
Vitamin D deficiency				
No	ref.	-	ref.	-
Yes	1.00 (0.75, 1.32)	0.977	1.02 (0.77, 1.37)	0.875
**Second trimester**				
25(OH)D, ng/mL	1.03 (0.93, 1.14)	0.546	1.06 (0.96, 1.18)	0.250
Vitamin D deficiency				
No	ref.	-	ref.	-
Yes	0.90 (0.55, 1.48)	0.681	0.85 (0.51, 1.43)	0.539
**Third trimester**				
25(OH)D, ng/mL	0.90 (0.79, 1.02)	0.110	0.95 (0.82, 1.10)	0.465
Vitamin D deficiency				
No	ref.	-	ref.	-
Yes	1.90 (1.03, 3.49)	0.040	1.54 (0.79, 2.99)	0.206

* Model 1: Crude model

† Model 2: Adjusted for maternal age, pre-pregnancy BMI, educational level, parity, PTB history, gestational week at blood sampling and sampling season

Table S2. Association of 25(OH)D concentrations in three trimesters with spontaneous PTB

Variables	Model 1*		Model 2†	
	OR (95% CI)	*p*	OR (95% CI)	*p*
**First trimester**				
25(OH)D, ng/mL	0.95 (0.84, 1.08)	0.460	0.94 (0.82, 1.07)	0.350
Vitamin D deficiency				
No	ref.	-	ref.	-
Yes	1.17 (0.77, 1.79)	0.465	1.24 (0.80, 1.91)	0.344
**Second trimester**				
25(OH)D, ng/mL	1.00 (0.85, 1.18)	0.987	0.99 (0.83, 1.18)	0.910
Vitamin D deficiency				
No	ref.	-	ref.	-
Yes	0.94 (0.42, 2.11)	0.878	0.97 (0.42, 2.24)	0.938
**Third trimester**				
25(OH)D, ng/mL	0.86 (0.71, 1.04)	0.111	0.91 (0.74, 1.12)	0.358
Vitamin D deficiency				
No	ref.	-	ref.	-
Yes	3.20 (1.40, 7.32)	0.006	2.69 (1.08, 6.68)	0.034

* Model 1: Crude model

† Model 2: Adjusted for maternal age, pre-pregnancy BMI, educational level, parity, PTB history, gestational week at blood sampling and sampling season

Table S3. Association of 25(OH)D concentrations in three trimesters with spontaneous PTB from MR analysis

Variables	n (%)	Model 1*		Model 2†	
		OR (95% CI)	*p*	OR (95% CI)	*p*
**First trimester**					
25(OH)D, ng/mL	103 (4.94)	1.00 (0.94, 1.06)	0.976	1.00 (0.95, 1.06)	0.995
Vitamin D deficiency					
No	33 (4.47)	ref.	-	ref.	-
Yes	70 (5.20)	1.00 (0.78, 1.30)	0.976	1.00 (0.79, 1.27)	0.995
**Second trimester**					
25(OH)D, ng/mL	33 (3.00)	1.02 (0.98, 1.06)	0.295	1.03 (0.98, 1.08)	0.270
Vitamin D deficiency					
No	25 (3.05)	ref.	-	ref.	-
Yes	8 (2.87)	0.88 (0.70, 1.12)	0.301	0.86 (0.64, 1.14)	0.280
**Third trimester**					
25(OH)D, ng/mL	23 (1.86)	1.00 (0.98, 1.03)	0.896	1.00 (0.98, 1.03)	0.947
Vitamin D deficiency					
No	11 (1.20)	ref.	-	ref.	-
Yes	12 (3.75)	0.99 (0.82, 1.19)	0.896	0.99 (0.83, 1.19)	0.947

* Model 1: Crude model

† Model 2: Adjusted for maternal age, pre-pregnancy BMI, educational level, parity, PTB history, gestational week at blood sampling and sampling season

Table S4. Association of 25(OH)D concentrations in three trimesters with PROM

Variables	Model 1*		Model 2†	
	OR (95% CI)	*p*	OR (95% CI)	*p*
**First trimester**				
25(OH)D, ng/mL	1.05 (0.99, 1.11)	0.082	1.05 (0.99, 1.12)	0.100
Vitamin D deficiency				
No	ref.	-	ref.	-
Yes	0.82 (0.67, 1.00)	0.055	0.83 (0.67, 1.01)	0.068
**Second trimester**				
25(OH)D, ng/mL	1.00 (0.95, 1.07)	0.878	1.01 (0.95, 1.08)	0.768
Vitamin D deficiency				
No	ref.	-	ref.	-
Yes	1.07 (0.81, 1.42)	0.637	1.05 (0.78, 1.42)	0.725
**Third trimester**				
25(OH)D, ng/mL	1.08 (1.03, 1.14)	0.003	1.06 (1.00, 1.12)	0.061
Vitamin D deficiency				
No	ref.	-	ref.	-
Yes	0.69 (0.50, 0.94)	0.020	0.78 (0.55, 1.09)	0.144

* Model 1: Crude model

† Model 2: Adjusted for maternal age, pre-pregnancy BMI, educational level, parity, gestational age, gestational week at blood sampling and sampling season

Table S5. Association of 25(OH)D concentrations in three trimesters with PPROM

Variables	Model 1*		Model 2†	
	OR (95% CI)	*p*	OR (95% CI)	*p*
**First trimester**				
25(OH)D, ng/mL	1.11 (0.95, 1.30)	0.173	1.10 (0.94, 1.30)	0.233
Vitamin D deficiency				
No	ref.	-	ref.	-
Yes	0.82 (0.46, 1.45)	0.498	0.87 (0.48, 1.57)	0.642
**Second trimester**				
25(OH)D, ng/mL	1.03 (0.85, 1.24)	0.796	1.05 (0.86, 1.29)	0.609
Vitamin D deficiency				
No	ref.	-	ref.	-
Yes	0.78 (0.29, 2.10)	0.618	0.71 (0.26, 1.98)	0.515
**Third trimester**				
25(OH)D, ng/mL	0.97 (0.75, 1.24)	0.797	0.99 (0.75, 1.30)	0.947
Vitamin D deficiency				
No	ref.	-	ref.	-
Yes	1.01 (0.27, 3.83)	0.987	0.86 (0.21, 3.61)	0.836

* Model 1: Crude model

† Model 2: Adjusted for maternal age, pre-pregnancy BMI, educational level, parity, PTB history, gestational week at blood sampling and sampling season

Table S6. Association of single SNP with PTB

Variables	Model 1*		Model 2†	
	OR (95% CI)	*p*	OR (95% CI)	*p*
rs1155563				
TT	ref.	-	ref.	-
TC	0.95 (0.71, 1.27)	0.732	0.94 (0.70, 1.26)	0.677
CC	0.94 (0.65, 1.38)	0.762	0.93 (0.63, 1.35)	0.693
rs16846876				
AA	ref.	-	ref.	-
AT	0.97 (0.74, 1.27)	0.808	0.96 (0.73, 1.26)	0.746
TT	0.73 (0.44, 1.19)	0.207	0.71 (0.43, 1.17)	0.182
rs2298849				
AA	ref.	-	ref.	-
AG	1.04 (0.78, 1.37)	0.810	1.05 (0.79, 1.39)	0.734
GG	1.15 (0.76, 1.72)	0.513	1.17 (0.78, 1.76)	0.453
rs7041				
AA	ref.	-	ref.	-
AC	0.85 (0.64, 1.13)	0.264	0.85 (0.64, 1.13)	0.273
CC	1.39 (0.87, 2.22)	0.173	1.44 (0.89, 2.30)	0.135
rs4588				
GG	ref.	-	ref.	-
GT	1.10 (0.84, 1.44)	0.490	1.08 (0.82, 1.41)	0.596
TT	0.54 (0.31, 0.93)	0.027	0.52 (0.30, 0.90)	0.020
rs2209314				
TT	ref.	-	ref.	-
CT	1.00 (0.75, 1.34)	0.992	0.99 (0.74, 1.33)	0.951
CC	1.18 (0.81, 1.72)	0.397	1.16 (0.79, 1.69)	0.456

* Model 1: Crude model

† Model 2: Adjusted for maternal age, pre-pregnancy BMI, educational level, parity and PTB history

Table S7. Association of single SNP with spontaneous PTB

Variables	Model 1[*]		Model 2[†]	
	OR (95% CI)	*p*	OR (95% CI)	*p*
rs1155563				
TT	ref.	-	ref.	-
TC	0.86 (0.55, 1.34)	0.505	0.88 (0.56, 1.36)	0.559
CC	1.10 (0.64, 1.90)	0.732	1.11 (0.64, 1.92)	0.712
rs16846876				
AA	ref.	-	ref.	-
AT	1.03 (0.68, 1.55)	0.898	1.03 (0.68, 1.55)	0.891
TT	0.77 (0.36, 1.64)	0.494	0.76 (0.35, 1.63)	0.474
rs2298849				
AA	ref.	-	ref.	-
AG	1.05 (0.68, 1.61)	0.825	1.05 (0.68, 1.62)	0.814
GG	1.22 (0.67, 2.20)	0.516	1.17 (0.65, 2.13)	0.597
rs7041				
AA	ref.	-	ref.	-
AC	0.84 (0.55, 1.28)	0.411	0.84 (0.55, 1.28)	0.422
CC	1.17 (0.55, 2.51)	0.680	1.24 (0.58, 2.67)	0.582
rs4588				
GG	ref.	-	ref.	-
GT	0.93 (0.62, 1.40)	0.740	0.93 (0.62, 1.40)	0.722
TT	0.58 (0.26, 1.28)	0.177	0.57 (0.26, 1.28)	0.175
rs2209314				
TT	ref.	-	ref.	-
CT	0.84 (0.54, 1.30)	0.425	0.80 (0.51, 1.25)	0.324
CC	1.22 (0.70, 2.10)	0.482	1.19 (0.69, 2.06)	0.530

[*] Model 1: Crude model

[†] Model 2: Adjusted for maternal age, pre-pregnancy BMI, educational level, parity and PTB history

Table S8. Association of single SNP with PROM

Variables	Model 1[*]		Model 2[†]	
	OR (95% CI)	*p*	OR (95% CI)	*p*
rs1155563				
TT	ref.	-	ref.	-
TC	0.94 (0.77, 1.16)	0.584	0.95 (0.77, 1.17)	0.623
CC	0.88 (0.67, 1.16)	0.361	0.87 (0.67, 1.15)	0.331
rs16846876				
AA	ref.	-	ref.	-
AT	0.85 (0.70, 1.03)	0.100	0.85 (0.70, 1.03)	0.100
TT	0.90 (0.65, 1.23)	0.500	0.90 (0.65, 1.24)	0.508
rs2298849				
AA	ref.	-	ref.	-
AG	0.90 (0.74, 1.10)	0.326	0.91 (0.75, 1.12)	0.382
GG	1.18 (0.89, 1.56)	0.257	1.18 (0.89, 1.57)	0.244
rs7041				
AA	ref.	-	ref.	-
AC	1.13 (0.93, 1.37)	0.212	1.13 (0.93, 1.37)	0.216
CC	1.25 (0.87, 1.80)	0.226	1.24 (0.86, 1.79)	0.251
rs4588				
GG	ref.	-	ref.	-
GT	0.87 (0.71, 1.05)	0.150	0.86 (0.70, 1.05)	0.131
TT	0.89 (0.65, 1.21)	0.461	0.90 (0.66, 1.23)	0.520
rs2209314				
TT	ref.	-	ref.	-
CT	1.13 (0.92, 1.39)	0.248	1.15 (0.93, 1.41)	0.202
CC	1.17 (0.89, 1.54)	0.270	1.15 (0.88, 1.52)	0.309

[*] Model 1: Crude model

[†] Model 2: Adjusted for maternal age, pre-pregnancy BMI, educational level, parity and gestational age

Table S9. Association of single SNP with PPROM

Variables	Model 1*		Model 2†	
	OR (95% CI)	*p*	OR (95% CI)	*p*
rs1155563				
TT	ref.	-	ref.	-
TC	0.86 (0.47, 1.56)	0.612	0.84 (0.46, 1.54)	0.582
CC	0.85 (0.39, 1.88)	0.688	0.84 (0.38, 1.86)	0.667
rs16846876				
AA	ref.	-	ref.	-
AT	0.77 (0.43, 1.37)	0.373	0.76 (0.43, 1.36)	0.353
TT	0.64 (0.22, 1.84)	0.408	0.63 (0.22, 1.81)	0.389
rs2298849				
AA	ref.	-	ref.	-
AG	0.90 (0.50, 1.61)	0.712	0.91 (0.50, 1.64)	0.751
GG	1.03 (0.44, 2.41)	0.951	1.05 (0.45, 2.46)	0.915
rs7041				
AA	ref.	-	ref.	-
AC	0.65 (0.34, 1.24)	0.195	0.65 (0.34, 1.24)	0.196
CC	2.60 (1.22, 5.55)	0.014	2.68 (1.25, 5.74)	0.011
rs4588				
GG	ref.	-	ref.	-
GT	0.70 (0.39, 1.26)	0.233	0.69 (0.38, 1.24)	0.210
TT	0.71 (0.27, 1.86)	0.491	0.70 (0.27, 1.82)	0.458
rs2209314				
TT	ref.	-	ref.	-
CT	1.25 (0.67, 2.33)	0.478	1.24 (0.67, 2.32)	0.494
CC	1.24 (0.54, 2.82)	0.608	1.23 (0.54, 2.80)	0.625

* Model 1: Crude model

† Model 2: Adjusted for maternal age, pre-pregnancy BMI, educational level, parity and PTB history
